# 

**Published:** 1870-07

**Authors:** 


### CONTENTS.

ORIGINAL COMMUNICATIONS.

Pathology ... 285
Hypodermic Medication in Dental Practice ... 299
Extraction of a Tooth ... 304
Treatment and Preservation of the Deciduous Teeth ... 306

PROCEEDINGS OF SOCIETIES.

The Fifth New York District Dental Society ... 312
Forest City Society of Dental Surgeons ... 316

SELECTIONS.

The Nitrie of Amyl ... 321
Effects of Tobacco-Smoking on Children ... 322
Stammering and Stutte ing ... 322
Deaths from Chloroform ... 323
Vertigo from Tobacco ... 323
Present Aspect of Medical Science—By N J. Davis ... 324

EDITORIAL.

The Cincinnati College of Medicine and Surgery ... 325
The Law—Explanatory ... 326
Casting Aluminum ... 327
The American Dental Association ... 327
A Request ... 328

